# First Study in Men Evaluating a Surgical Robotic Tool Providing Autonomous Inner Ear Access for Cochlear Implantation

**DOI:** 10.3389/fneur.2022.804507

**Published:** 2022-03-21

**Authors:** Vedat Topsakal, Emilie Heuninck, Marco Matulic, Ahmet M. Tekin, Griet Mertens, Vincent Van Rompaey, Pablo Galeazzi, Masoud Zoka-Assadi, Paul van de Heyning

**Affiliations:** ^1^Department of Otorhinolaryngology Head and Neck Surgery, University Hospital UZ Brussel, Vrije Universiteit Brussel, Brussels, Belgium; ^2^CASCINATION AG, Bern, Switzerland; ^3^Department of Otorhinolaryngology, Klinikum Bad Salzungen, Bad Salzungen, Germany; ^4^Department of Otorhinolaryngology, Head and Neck Surgery, Department of Translational Neurosciences, Faculty of Medicine and Health Sciences, Antwerp University Hospital, University of Antwerp, Antwerp, Belgium; ^5^MED-EL Medical Electronics, Innsbruck, Austria

**Keywords:** sensorineural hearing loss (SNHL), cochlear implantation, image-guided surgery, robotically-assisted cochlear implantation surgery, HEARO procedure

## Abstract

Image-guided and robot-assisted surgeries have found their applications in skullbase surgery. Technological improvements in terms of accuracy also opened new opportunities for robotically-assisted cochlear implantation surgery (RACIS). The HEARO^®^ robotic system is an otological next-generation surgical robot to assist the surgeon. It first provides software-defined spatial boundaries for orientation and reference information to anatomical structures during otological and neurosurgical procedures. Second, it executes a preplanned drill trajectory through the temporal bone. Here, we report how safe the HEARO procedure can provide an autonomous minimally invasive inner ear access and the efficiency of this access to subsequently insert the electrode array during cochlear implantation. In 22 out of 25 included patients, the surgeon was able to complete the HEARO^®^ procedure. The dedicated planning software (OTOPLAN^®^) allowed the surgeon to reconstruct a three-dimensional representation of all the relevant anatomical structures, designate the target on the cochlea, i.e., the round window, and plan the safest trajectory to reach it. This trajectory accommodated the safety distance to the critical structures while minimizing the insertion angles. A minimal distance of 0.4 and 0.3 mm was planned to facial nerve and chorda tympani, respectively. Intraoperative cone-beam CT supported safe passage for the 22 HEARO^®^ procedures. The intraoperative accuracy analysis reported the following mean errors: 0.182 mm to target, 0.117 mm to facial nerve, and 0.107 mm to chorda tympani. This study demonstrates that microsurgical robotic technology can be used in different anatomical variations, even including a case of inner ear anomalies, with the geometrically correct keyhole to access to the inner ear. Future perspectives in RACIS may focus on improving intraoperative imaging, automated segmentation and trajectory, robotic insertion with controlled speed, and haptic feedback. This study [Experimental Antwerp robotic research otological surgery (EAR2OS) and Antwerp Robotic cochlear implantation (25 refers to 25 cases) (ARCI25)] was registered at clinicalTrials.gov under identifier NCT03746613 and NCT04102215.

**Clinical Trial Registration:**

https://www.clinicaltrials.gov, Identifier: NCT04102215.

## Introduction

Since the introduction of direct electrical stimulation of the human auditory system more than 60 years ago, cochlear implants (CIs) are now widely regarded as one of the most successful neural prostheses in the modern world of otology (1). Thanks to the advances made in the field of biomedical engineering, the implants that are on the market today are able to bypass the damaged sensory hair cells in the cochlea of patients with severe-to-profound sensorineural hearing loss (SNHL). By exciting subpopulations of the auditory nerve directly with electrical pulses, CIs have restored hearing in more than 600,000 patients (2)^1^ The CI indication field is expected to expand significantly together with the growing and aging world population. Since an increased number of studies have showed a link between hearing loss and cognitive decline (3), hearing restoration will become increasingly important for people's health and well-being (4, 5). Moreover, the CI market is driven by an established history of successful technological innovation. Most innovations related to the reliability of the device (6), the design of the implant and electrode array (7), the miniaturized digital processing chips, and the speech coding strategies (8). Less research and, therefore, fewer modifications were made on the side of the surgical implantation techniques. In 1976, House first described the essential surgical steps for CI, including opening the skin flap, preparing the subperiosteal pocket, drilling the mastoidectomy and the facial recess approach (also called posterior tympanotomy), opening the scala tympani, inserting the electrode array, and fixating the implant (9). Until today, the facial recess approach is considered the golden standard, with a consistent rate of <1% of facial nerve (FN) injury. These cases most frequently are partial weaknesses of short duration or delayed-onset pareses, which resolve over time. In the past, alternative techniques to the facial recess approach have been suggested, such as the Veria (10), the suprameatal (11), and the pericanal approach (12). Although these approaches will reduce drilling near the FN, they have their own set of disadvantages such as difficult and traumatic insertion angles for the array, perforation of the tympanic membrane, and postoperative infection (13). Most subsequent innovations were concentrated on techniques to approach the scala tympani (14), adjusted techniques for the (partially) ossified cochlea (15) or dysplasia (16), techniques for hearing and structure preservation (17), intraoperative guiding recordings (18), and the use of corticosteroids (19).

The development of robotically-assisted cochlear implantation surgery (RACIS), therefore, has been evaluated in preclinical studies in the last decade and Labadie et al. succeeded in a clinical study for the first time (20–22). A new milestone was reached when Caversaccio et al. achieved the facial recess approach using the self-developed OtoBot with its own navigation (23). Robotic-assisted techniques have found their way to otological and neurosurgical procedures, which offer new possibilities for minimally invasive keyhole CI surgery. These techniques enable a tool position and orientation based on image data and virtual anatomical models to be calculated and visualized by the surgeon. Stereotactic navigation or the use of an image-based template was initially investigated in the context of CI surgery with the premise of replacing the traditional mastoidectomy in favor of a small tunnel drilled in a predetermined location with the aid of a navigation system (24, 25). In these early studies, researchers hypothesized that high navigation accuracies (tool positioning), typically <0.5 mm (26), were necessary to safely preserve critical anatomical structures [FN, chorda tympani (ChT), and ossicles]. Probably to adequately target specific areas of the cochlea [round window (RW)] for electrode insertion, even a higher accuracy is necessary. Ultimately, stereotaxy alone was unable to achieve sufficient control of the tool position relative to anatomical locations due to insufficient accuracy of the navigation system itself and lack of a mechanical tool positioning method to overcome the limitations of human dexterity. Further research sought to develop the use of mechanical positioning devices such as patient-specific templates (27, 28) and robotic manipulators (29, 30). Each of the previous designs possessed insufficient accuracy; thus, no stereotactic aid has been routinely used in otological surgery.

The OtoBot robotic system was developed to achieve the goal of a tunnel-based direct robotic middle ear access (31). The feasibility of the robotic middle ear access through the facial recess was successfully demonstrated in 6 patients using the OtoBot system at Insel Hospital, Bern in Switzerland (ClinicalTrials.gov Identifier: NCT02641795). However, the surgeon created the inner ear access manually through the ear canal after lifting the tympanomeatal flap. The electrode array insertion was also performed manually into the cochlea through cochleostomy (31). When middle ear access is not perfectly aligned with inner ear access, it is surgically very challenging to insert a flexible array, since there is little space for manipulations. Aligned in this sense means literally the continuation of the inner ear access in the same line as the middle ear access. Therefore, the next phase of the development of robotic workflow focused on the robotic inner ear access thought the already gained keyhole access through mastoid. This step also implies a significant contribution toward structure and hearing preservation. Although this is a different aim and involves also a biological factor that cannot be controlled, robotic approaches aim to control the reduction of mechanical and noise-induced trauma and rupture of the RW membrane. Future robotic insertion should aim to avoid intracochlear pressure disturbances causing damage and possibly hearing deterioration.

The current RACIS given by the HEARO procedure also meets the demand for more accuracy and provides a new robotic inner ear diamond burr that is an equivalent of conventional microdrills (30–32). For the middle ear access, a 1.8-mm drill needs to pass through the facial recess that has an average size of 2.54 ± 0.5 mm (23), which is already very demanding for a systems accuracy in terms of safety. In inner ear access, there is even less room for inaccuracy because the 1.0 mm diameter diamond burr needs to be rather perfectly aligned with the RW membrane with a crucial diameter of 1.31 ± 0.31 mm (33). RACIS needs to provide even more accuracy in terms of successful insertion in the scala tympani aligned with the basilar membrane (34, 37).

The main objectives of this first-in-man clinical trial using the HEARO were:

- To evaluate the intraoperative accuracy of robotic middle ear and inner ear access with regard to the distance to critical anatomical structures (such as the ChT and FN) and the designated target (i.e., opening the bony overhang of the round window niche or *canonus* and targeting the center of the round window membrane).
- To evaluate whether full manual insertion of the electrode array could be achieved through the drilled tunnel.

## Materials and Methods

### Study Design

We performed an interventional clinical trial in two stages. First, a pilot study for the feasibility of RACIS including access to the inner ear was completed for the first time in men. This study (EAR2OS) was registered at clinicalTrials.gov under identifier NCT03746613 and the HEARO device exemption number 80M0763 from the Belgium Competent Authority [Federal Agency for Medicines and Health Products (FAMHP)]. The approval of the Antwerp University Hospital ethics committee was granted with number B300201837507. In a second stage, a follow-up pivotal study (ARCI25) was also performed involving the effectiveness of RACIS and registered under identifier NCT04102215. The approval of the Antwerp University Hospital ethics committee was granted with number B300201941457 and the HEARO device exemption 80M0793. Adult (18 years or older) patients, running for cochlear implantation according to local reimbursement and candidacy criteria, were clinically and radiologically screened for eligibility. All the participants gave a written informed consent to the same ear, nose, and throat (ENT) surgeon counseling them and performing all the surgeries (VT). The inclusion criteria comprised adult CI candidates with suitable anatomy opting for a Medical Electronics (MED-EL) device. Patients for instance with previous temporal bone surgery, e.g., radical cavities were excluded. Exclusion criteria consisted of pregnancy, the vulnerability of the patient (not able to consent), and withdrawn or invalid informed consent. Radiological exclusion criteria were defined by a planned trajectory on the routine clinical high-resolution CT (HRCT) scan often using 0.3 mm slice thickness: a distance to FN <0.4 mm and <0.3 mm to ChT were excluded from this study.

### HEARO Procedure

**Figure 1:**
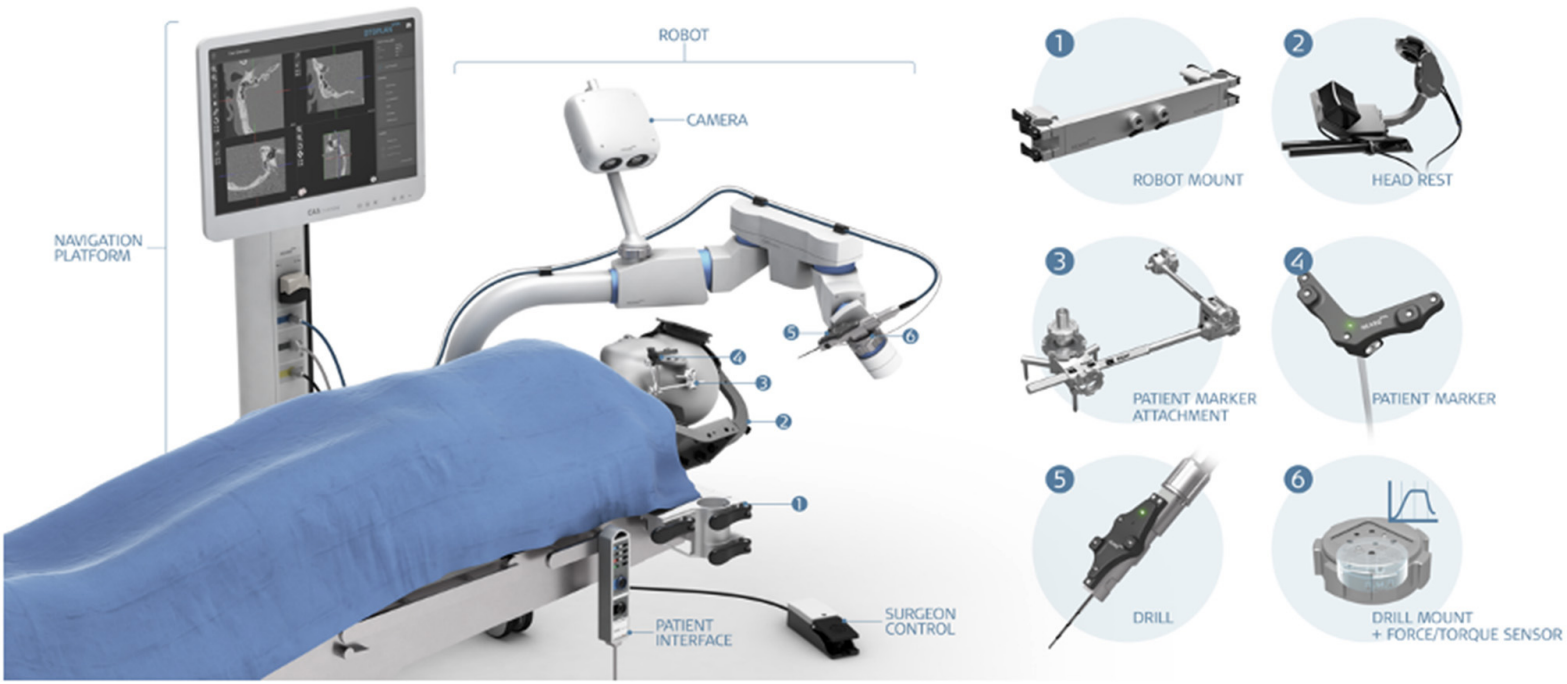
The HEARO^®^ robotic system. **(1)** Robot mount, **(2)** headrest, **(3)** patient marker attachment, **(4)** patient marker, **(5)** drill, and **(6)** drill mount with force/torque sensor.

**Figure 2:**
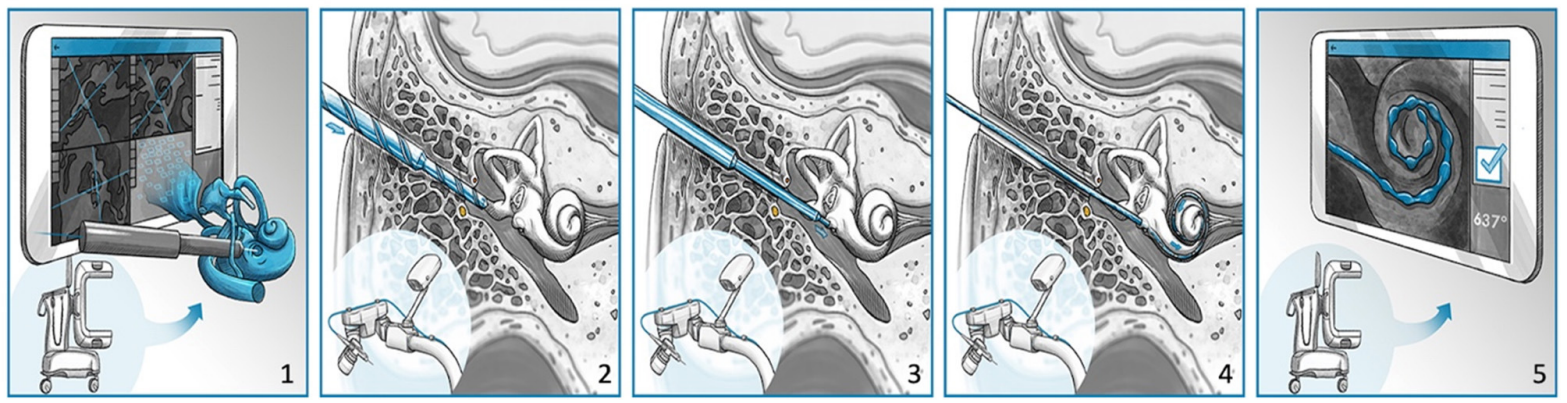
The HEARO procedure for cochlear implantation surgery. **(1)** Scanning and planning, **(2)** performing middle ear access with cutting bur, **(3)** performing inner ear access with diamond bur, **(4)** placement of array through a removable insertion tube, and **(5)** postoperative scanning and quality analysis.

The HEARO^®^ robotic system (CASCINATION AG, Bern, Switzerland) is an assistive otological next-generation surgical robot (Figure 1). It integrates a set of sensors, actuators, and core functionalities to allow the surgeon to perform image-guided surgery with a robotic arm. The HEARO procedure for CI surgery is described below and comprises three main stages followed by postoperative analyses (Figure 2). Today, the current HEARO system is “conformité européenne (CE)” marked for clinical use in adults and requires a minimal planned distance of 0.4 mm to the FN (26). In preoperative analyses of patients, it is possible to estimate the cochlear duct lengths (CDLs) for tailored and complete cochlear coverage for an optimal audiological outcome (36). In this study, patients were inserted with a 28-mm (96%) and a 20-mm (4%) electrode array (MED-EL, Innsbruck, Austria). Both the electrodes have a diameter of 0.8 mm and allow, therefore, passage to middle ear by the 1.8 mm tunnel and access to inner ear by the 1.0 mm diameter diamond burr.

A commercially available mobile cone-beam CT (CBCT) with 0.1 mm spatial resolution (XCAT XL, Xoran Ltd, Ann Arbor, Michigan, USA) acquired radiological images for preoperative planning, checking the partially drilled trajectory, and for postoperatively checking the array placement in the cochlea. Visual inspection of the cochlear entrance site was assured by a conventional microscope for stereoscopic viewing through the ear canal allowing the surgeon to work with both the hands or an endoscope that could view closer to the target, but always occupied one surgical hand. This step may become obsolete in future protocols, but in this study visual inspection served as a safety control. Also, commercially available multichannel endoscopes with a diameter of 1.3 mm (Carl Storz, Denzlingen, Germany) allowed the surgeon for visual inspection, irrigation, and suction through the drilled tunnel when desired.

#### Preparations and Planning

The patients were prepared for surgery and the head was non-invasively immobilized into a customized head clamp in slight hyperextension of the neck and rotation to the contralateral side. After a retroauricular incision, five fiducial screws (four for image to patient registration and one for patient marker attachment) were placed on the mastoid cortex, as artificial landmarks before the preoperative imaging were required for subsequent navigation. Then, the patient's head was scanned with the mobile CBCT (0.1 mm resolution) and the images were imported to the dedicated planning software (OTOPLAN^®^, CASCINATION AG, Bern, Switzerland). The surgeon three-dimensionally reconstructs all the relevant anatomical structures of the FN, the ChT, the ossicles, and the external auditory canal. The surgeon then manually sets the target point at the level of the RW membrane and adjusts the ideal trajectory line based on the patient's specific anatomy and in-plane and out-plane angels, as previously described by Wimmer et al. (34). Sufficient safety distances and individualized inner ear access are optimized. The surgical plan is exported from OTOPLAN to the HEARO. The HEARO software automatically renders, if the planned trajectory is executable within the safety margins to the FN. The surgeon performs a patient-to-image registration to enable the navigation of the robot. Possible planning out of the reach of the robot arm or possible collision with fiducials are signaled to the surgeon by the system.

#### Performing Robotic Drilling

In this stage, the robotic arm executes the surgical plan. With a custom-made helical step drill of 1.8 mm diameter, the first access is drilled into the middle ear, which is decomposed in three phases:

(i) Drilling from the cortex of mastoid bone until 3 mm before the level of the FN.

(ii) Drilling through the facial recess.

(iii) Drilling mastoid cells further than FN to complete the middle ear access.

After completion of phase (i), a titanium rod is placed in the partially drilled tunnel by the surgeon and intraoperative imaging was performed in every case. The rod enhances the contrast of the drilling tunnel in the image. The image is loaded into the planning software allowing the surgeon to the assessment of the safety margins between the drilling trajectory and the anatomy as well as for the measurement of the drilling accuracy. Upon confirmation of the safe trajectory (not compromising FN), the drilling can be continued through the facial recess at phase (ii). Here, multipolar FN stimulation would be performed five times at 0.5 mm intervals providing diverse and from navigation independent means to verify the safe distances to the FN. Phase (iii) is usually swiftly performed because it is beyond FN and not near middle ear structures. In some cases, there is also very little bone left here to drill. Throughout cortical drilling toward the middle ear, the robotic drilling is performed in pecking cycles. The drill bit needs to come out of the trajectory for automatic irrigation to clean the helix of the drill bit. It also allows the surgeon to clean the drilled tunnel and check for possible bleeding tendencies. The cleanness of the drill bit is necessary to avoid extensive heat to FN during drilling (26).

##### Inner Ear Access

**Figure 3:**
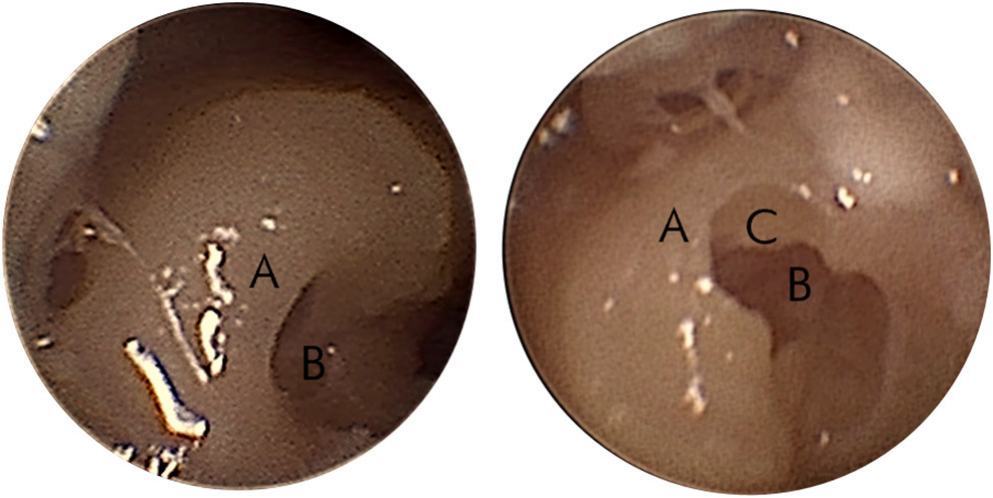
Endoscopic view of the partial canonostomy. An example of the endoscopic view of the canonus **(A)** and the round window (RW) niche **(B)** on the left side. The right side shows a partial canonectomy **(C)** during intraoperative surgical check.

**Figure 4:**
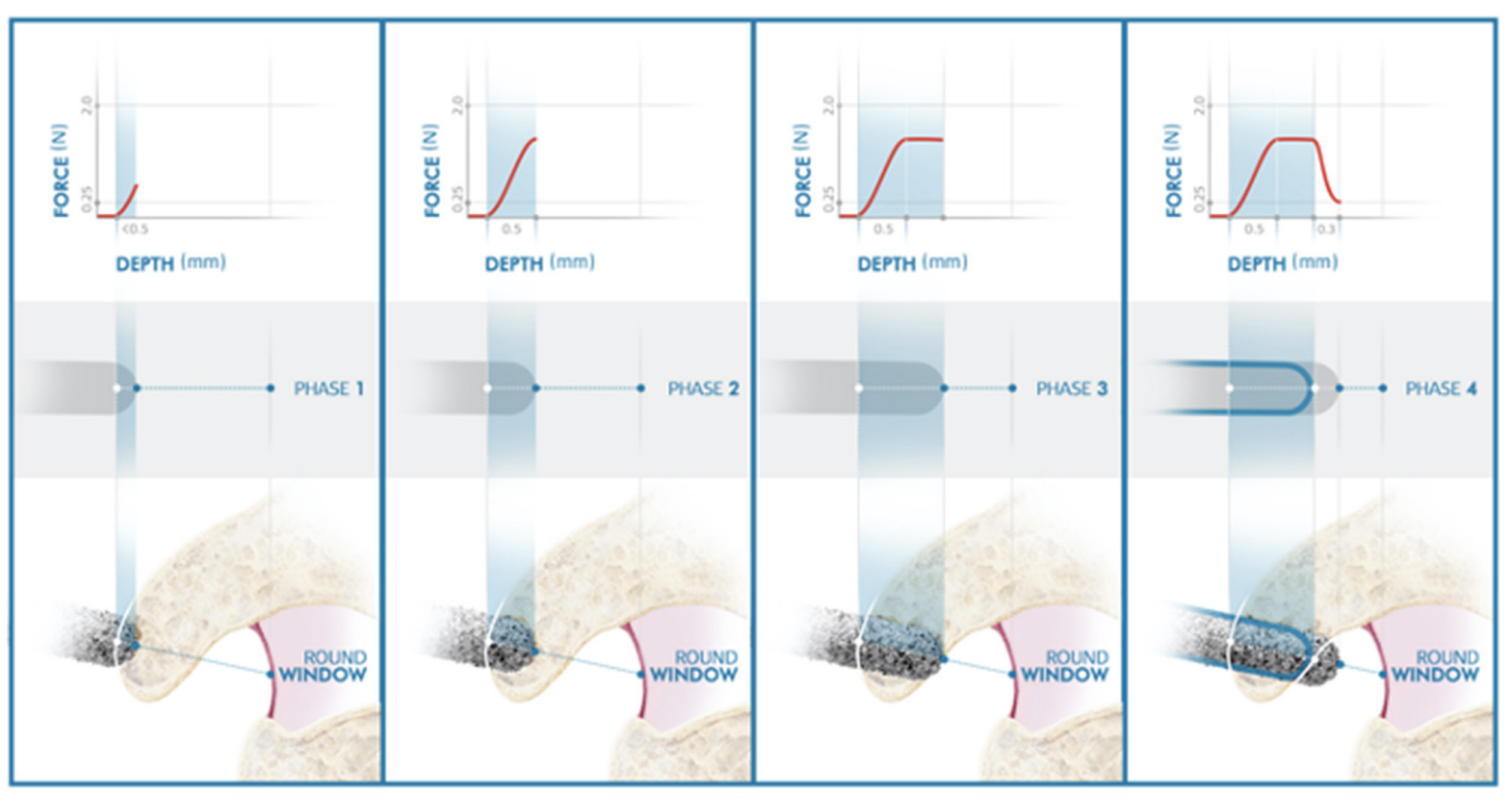
Illustration of the inner ear access of the HEARO. The distance between point lateral wall (LW) and medial wall (MW) represents the bone thickness of the inner ear access point. The red rectangle under the graph also represents the thickness of the bony wall. The white solid line on the graph defines the target point set by the user at the preoperative planning stage. The filled blue line represents the force transients and the exact force at each specific point and is also displayed inside the burr illustration under the graph. The dashed white line represents the estimated point at which the size of 0.9 mm for the opening is achieved.

After completion of the middle ear access, a 1.0-mm tungsten-carbide diamond burr with fine diamond coating needs to be correctly mounted for milling a canonostomy, which is a hole in the bony overhang of the round window (37). The inner ear access is achieved by combining preoperative and intraoperative parameters. The canonus thickness was predicted preoperatively and milling forces from the six-axis force-torque sensor of the arm (Mini-40, SI-20-1 calibration, ATI, USA) and intraoperative depth of the drill in the trajectory was intraoperatively determined from the navigation of the system. As the milling starts, the surgeon observes and follows the force graph on the robot interface to determine the relative position of the diamond burr in respect of the canonus (38, 39). The tunnel approach for millimetric keyhole surgery limits the visual feedback for the surgeon during the drilling of the canonus. Particularly, the depth of the burr tip cannot be continuously assessed by vision. Therefore, the surgeon has to mainly rely on the information provided by the system graphical user interface (GUI) instead of visual feedback. This situation without surgical view is out of the surgeon's comfort zone. It is important to note that the surgeon can stop the milling at any point and visually inspect the access either through the drilled keyhole exposition with endoscope or with the microscope through the ear canal by lifting the eardrum. An example of an endoscopic view over the partial canonostomy is shown in Figure 3. This maneuver of lifting the eardrum is also necessary for electrode insertion later on and is a rather standardized procedure for a trained otologist. After a stop, the system allows it to continue according to surgical plan or to further mill a selectable distance. To avoid damage to the inner structures of the cochlea, drilling cannot be further than the target point. If a surgeon suspects a target point was set wrongly, it is even possible to abort from RACIS and continue manually. Figure 4 illustrates the inner ear access algorithm of the newest generation RACIS with the HEARO.

Canonostomy can be divided into four phases depending on the location of the diamond burr.

Phase I: The diamond burr reaches the lateral wall (LW) of the canonus and the force profile starts increasing with a steep gradient: **the touchdown phase**. The depth of the LW of the canonus predicted in the preoperative planning is used as an estimation depth at which the contact should occur. In an ideal case, phase I shall start exactly at the estimated LW point in the preoperative planning. However, the surgeon needs to verify this, so the inner ear algorithm is not starting prematurely. If the force profile rises before or after the estimated LW point, the surgeon can shift the preset LW line. This would automatically shift the medial wall (MW) line to maintain predicted bone thickness of canonus.

Phase II: The diamond burr is fully in the canonus: **the plateau phase**. If the canonus is sufficiently thick and approached rather perpendicularly, as simulated in Figure 4, the force profile stabilizes. Perpendicular angles on canonus are likely to have a plateau phase, whereas more tangential angles may not. The latter represent a grazing shot on canonus. There is automated feedback between the milling speed and feedforward rate. When a force threshold of 2.0 N is applied by the system and when this force is reached, the system automatically adjusts the feed rate to reduce the milling force.

Phase III: The diamond burr just reached the MW of canonus**: the breakthrough phase**. As bone in front of the drill becomes very thin, it begins to deform locally resulting in a drop of force. The MW selected in the preoperative planning is used as an estimation at which the breakthrough occurs, but again the surgeon is observing the force graph and must confirm this moment.

Phase IV: The diamond burr is in the RW niche: **the enlarging phase**. The diameter of the CI array used here is 0.8 mm. For a frictionless passage, the minimum required size of the canonostomy is 0.9 mm. Since the diameter of the drill burr is 1 mm, a further 0.3 mm milling after the MW is required to achieve this. The system automatically stops the milling process at the predicted target depth. The surgeon needs to verify, if this predicted target is correct and if an insertion is possible. When a larger canonostomy is desired, the surgeon also needs to verify that there is enough distance between the MW of canonus and the RW membrane in the RW niche for that specific trajectory.

#### Placing the Array and Postoperative Analyses

The next step is the most critical for the aim of this study, but also for the aim of the surgery: correct placement of the array will determine the success of the surgery for the patient. Since the surgeon now has to take over from the robotic system, a visual exposure through the ear canal becomes indispensable. An insertion tube, consisting of two half-pipes, has to be placed in the drilled trajectory to avoid a false route of the array into aerated mastoid cells in the temporal bone. The insertion tube consists of two pieces, allowing for its removal from the drilled tunnel alongside the array after insertion and leaving the array in place. Furthermore, the insertion tube has been designed with a step to avoid overinsertion and the surgeon decides how far it may be inserted by selecting a target. The two composing pieces are of different lengths, allowing the surgeon to see how the array slides through toward the inner ear. The shorter piece has to be oriented toward the visualizing modality, either an endoscope or a microscope through the ear canal. Before insertion, the surgeon will have to perform classical steps of CI surgery according to local or personal habits. The surgeon will make an implant bed or will use other fixation methods: as such for the PIN implants that require a tight periosteal pocket and two pin holes drilled in the cortex of the temporal bone. The surgeon needs to position his or her hands to manually open the RW membrane, insert the electrode array, and take the insertion tube out the keyhole trajectory in two pieces without manipulating the array. Some standard surgical steps have to be performed as well: such as suturing the skin, sealing the RW area depending on the surgeon's preferences, and sinking the complete array into a cortical bone channel to protect against trauma. All these steps require some training for the surgeon to get acquainted with the view and the handlings.

A postoperative radiological CBCT image after the final stage of the procedure is performed to analyze the electrode's insertion status into the inner ear. Also, the planning software has postoperative analysis features that can even provide a processing strategy for the implant. Usually, electrophysiological tests can also confirm correct placement by registering impedance and currents of the implant to verify its functioning. The ultimate proof of a good placement is of course testing the hearing function and a symmetric smile on the patient.

Surgical follow-up included an overnight stay and evaluation of possible clinical complications in short term, but also in the long term with almost 1-year follow-up for all the participants. The primary aim of this study was to evaluate the effectiveness in terms of how many insertions were possible (and to what depth) with this protocol for RACIS and second to evaluate the safety in terms of the relative risk for FN damage or other complications.

## Results

### Demographics, Preparations, and Planning

**Figure 5:**
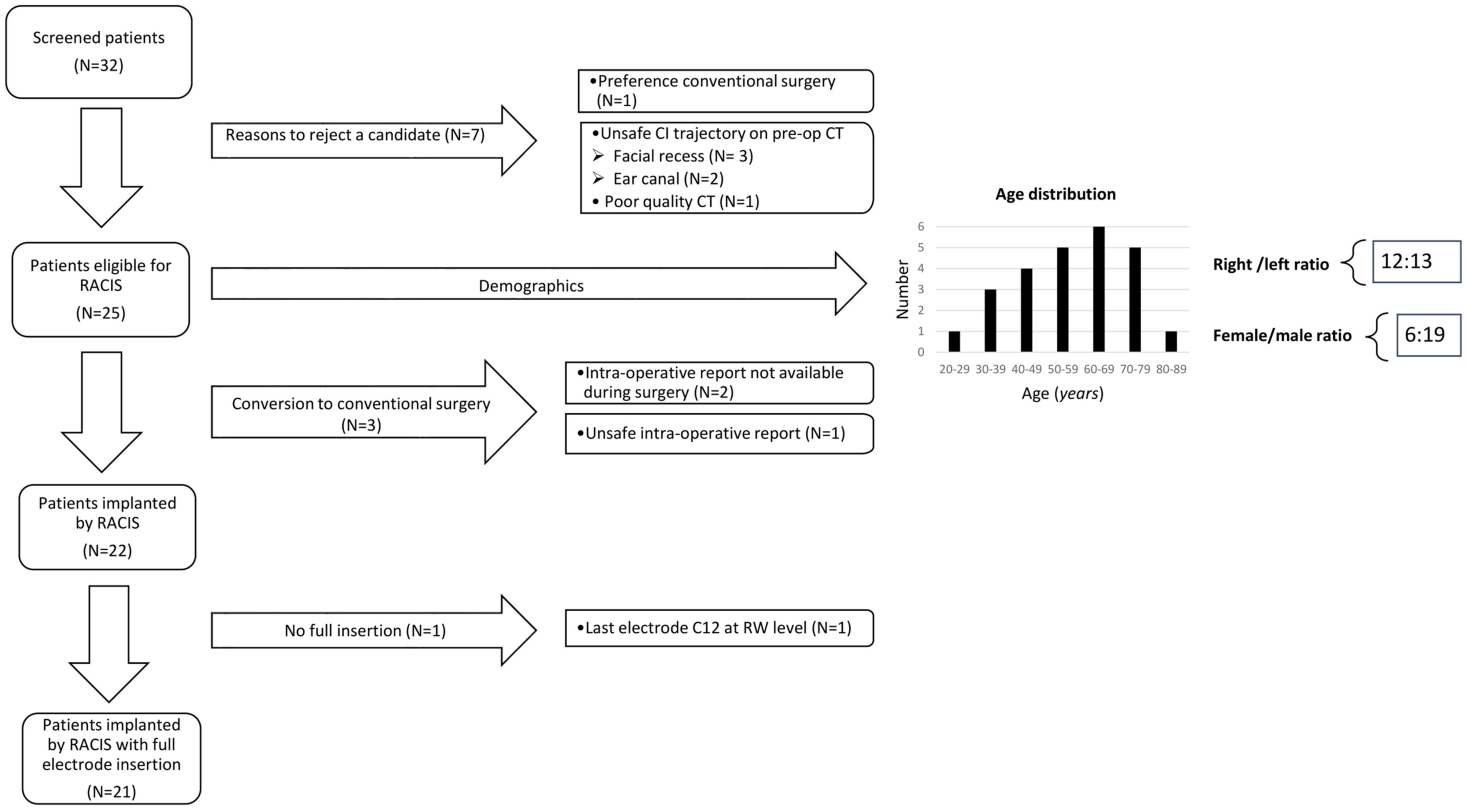
Patient selection and demographics.

All the eligible patients indicated for CI between December 2018 and July 2020 were checked for eligibility and 32 preoperative HRCT scans were screened. In three cases, the trajectory planning did not allow a safe trajectory because the distance to FN was smaller than 0.4 mm; in two cases, the surgeon decided that the distance to the ear canal is too and in one case, the ChT was not visible due to low soft-tissue contrast in the image. Thus, 26 segmented trajectories were safe according to this study protocol for RACIS. In total, 25 patients gave an informed consent and only one candidate chose to have the surgery in a conventional manner. The same surgeon did all the 25 cases. In 22 cases, the HEARO procedure was completed and in 3 cases, it had to be converted to conventional surgery and every patient indicated for CI received a CI (Figure 5). The age of the 25 participants ranged from 28 to 83 years; the study population consisted of 6 women (24%) and 19 men (76%) and the left-right ratio was 12:13. Evaluation of the inner ear anatomy of 21 patients showed normal CT scans. Three patients had ossification anomalies: one patient had a postmeningitis ossification, one patient had far advanced otosclerosis causing also (de)calcifications, and one patient with Cogan syndrome had an intracochlear calcification. One patient had an inborn genetic error and showed an incomplete partition type III (IP-III) anomaly of the inner ear (40). Table 1 shows a complete overview of the other etiologies of SNHL. When the etiology is marked as unknown, it usually involved progressive SNHL and table reports the onset of the hearing loss, but also the duration of hearing loss.

**Table 1:** Subjects' demographics.

**Subjects**	**Etiology**	**Implantation ear (R, right; L, left)**	**Age at implantation (years)**	**Age of hearing loss onset (years)**	**Inactive electrodes**
EAROS_1	Unknown	R	47	40	/
EAROS_2	Unknown	L	61	47	/
EAROS_3	*DFNA9*	R	56	49	/
ARCI25_1	Unknown	R	62	47	/
ARCI25_2	IP-III	R	71	46	/
ARCI25_3	Meningitis	L	56	6	/
ARCI25_4	Sudden SSD	L	47	45	/
ARCI25_5	(Neuro) Sarcoidosis	L	39	34	/
ARCI25_6	Usher	R	58	0	/
ARCI25_7	Sudden deafness	L	83	72	/
ARCI25_8	*DFNA9*	L	53	39	/
ARCI25_9	Unknown	R	42	39	/
ARCI25_10	*OPA1* mutation	R	38	12	/
ARCI25_11	*DFNA9*	R	68	40	/
ARCI25_12	Far advanced otosclerosis	L	56	39	/
ARCI25_13	Unknown	L	40	15	/
ARCI25_14	Unknown	R	76	71	e12
ARCI25_15	Unknown	L	75	12	/
ARCI25_16	Chronic middle ear infection	R	68	62	/
ARCI25_17	Unknown	L	70	50	/
ARCI25_18	Unknown	L	67	62	/
ARCI25_19	Unknown	L	64	53	/
ARCI25_20	Unknown	R	28	0	/
ARCI25_21	MELAS	R	62	50	/
ARCI25_22	Cogan syndrome	L	31	28	/
ARCI25_23	Unknown	R	78	58	/

**Figure 6:**
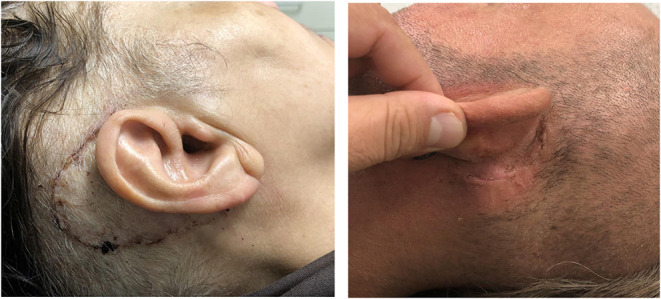
Retroauricular incision. Left side is one of the first 3 cases (initial EAR2OS trial) and right side all other more recent cases (ARCI25 trial).

In the initial EAR2OS trial, it took longer to prepare the surgical field with sterile draping and the incisions may have been more posterior. As more patients underwent the surgery, the scrub nurse and surgeon worked out a faster workflow to drape the patients and the incision was reduced to a standard retroauricular incision (Figure 6). The software for segmenting the anatomy and planning trajectories worked well as well as the patient-to-image registration. The signaled collisions of the robot or trajectories beyond the reach of the robot arm could be resolved by adjusting the patient and robot positioning or by repositioning the patient marker.

### Performing the Drilling

In three cases of the 25, RACIS was converted to conventional surgery because of the safety protocol for middle ear access. Therefore, the inner ear access in these cases was not even performed with assistance of the system. Although in all the cases, the electrophysiological safety checks for the FN respected our safety protocol. The reason of aborting RACIS was the intraoperative CT scan. In two cases, the intraoperative accuracy of the drilling trajectory could not be confirmed by OTOPLAN due to metal artifact or insufficient image contrast resolution and the procedure was converted to conventional. When the intraoperative scan was evaluated later on another computer, it demonstrated accuracy within safe margins for one case. In another case, where the software worked fine, the surgery was converted because intraoperative accuracy predicted a trajectory that was closer than 0.4 mm to FN. Concerning middle ear access, the accuracy of the RACIS procedures has been safe to our protocol in 22 out of 25 cases. In none of the cases the facial monitor gave a warning and there was no postoperative facial weakness or facial palsy (Table 2).

**Table 2:** Intraoperative accuracy.

**Variable**	**Mean (SD)**	**Median**
Stapes (mm)	0.183 (0.265)	0.078
Incus and malleus (mm)	0.097 (0.68)	0.096
External auditory canal (mm)	0.127 (0.110)	0.091
Facial nerve (mm)	0.117 (0.109)	0.091
Chorda tympani (mm)	0.107 (0.103)	0.082
In-plane (°)	0.239 (0.173)	0.225
Out-plane (°)	0.182 (0.159)	0.146
Entrance (mm)	0.127 (0.067)	0.124
Target (mm)	0.182 (0.124)	0.157

The inner ear access was completed in all the 22 cases according to protocol. It proved to have a steep learning curve for the surgeon to follow and rely on the information provided by the system GUI instead of visual feedback. In some cases, with suitable anatomy, the canonostomy performed by RACIS was filmed through the ear canal by a microscope or even an endoscope just to have visual feedback. The manipulations of the eardrum have led to an eardrum perforation in case ARCI25_11, due to the obligatory lifting of it for exposure in RACIS protocol and because of a desire for maximum visual inspection. It was immediately supported with temporalis muscle fascia in underlay and it showed uncomplicated healing in the follow-up. In three cases, a cochleostomy was planned from the beginning of RACIS. The reasons for cochleostomy were ossification of the RW in case ARCI25_3 and ARCI25_12 and better gusher management *via* cochleostomy through the ear canal in case ARCI25_2. This case, with an inner ear malformation, is reported in more details elsewhere by Tekin et al. (40). In case ARCI25_14, the smallest RW membrane measured 0.5 mm (on the screening CT) and an enlarged RW approach was drilled robotically.

### Placement of the Array and Postoperative Analyses

**Figure 7:**
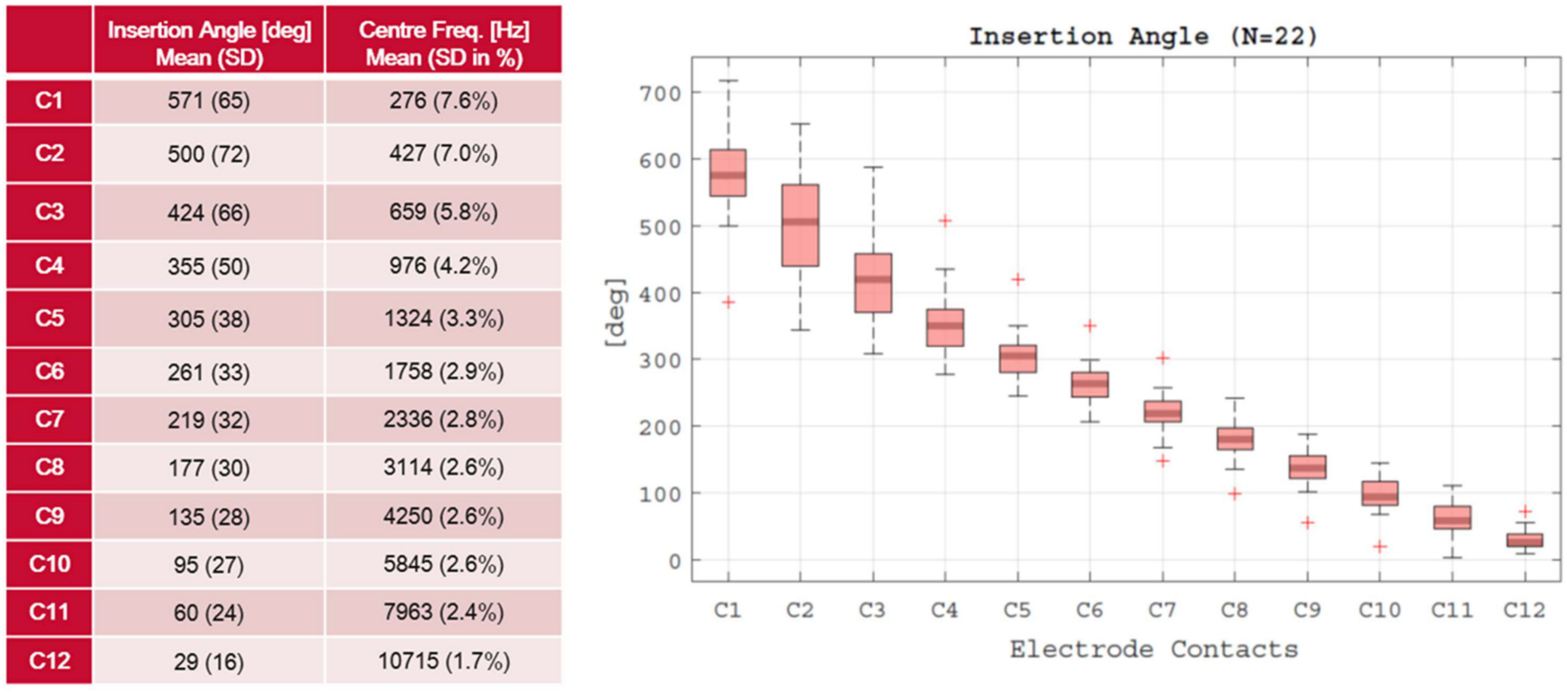
Insertion status.

The insertion tube proved very efficient in all the 22 cases after inner ear access was created successfully. It proved not only to be useful, but also easy to remove after insertion and removal did not affect the array once inserted. In 3 cases, the insertion of the array was not possible in the first effort. In case ARCI25_22, affected by Cogan syndrome, an intracochlear ossification hindered the first attempt. Full insertion, in this case, was only possible after a so-called Rambo technique (41) by widening the inner ear space with an insertion test device (ITD). In the case of ARCI25_5 and ARCI25_14, correct insertion of all the electrodes was also not possible in the first attempt. There was little space through the ear canal, but, moreover the angle to manipulate the array in a non-traumatic manner was very challenging. It was decided to robotically drill an enlarged RW approach in both the cases. Hereafter, full insertion was achieved for case ARCI25_5, but for case ARCI25_14, the angle of insertion still remained problematic and contact C12 could not be inserted further than where the RW membrane was. This was the only contact that remained outside in the complete series of 22 cases (Figure 5). Although the patient had an auditory sensation on this contact, it was switched off. The audiological results are not within the aim of this study and will be reported elsewhere. However, there was not a single electrode damaged in the complete study population including the converted cases. The insertion depth of electrodes is shown in Figure 7.

The postoperative CBCT not only allowed evaluation of the angular insertion depth, but confirmed placements in the scala tympani in all the 22 patients. Moreover, no tip fold-over could be identified on imaging. In 18 out of 22 cases, these scans also allowed to visualize the implant house handling in terms of fixation (PIN holes) and arrays embedded in channels. The distance from the keyhole access to the implant house was 35.1 mm (SD 8.4 mm) allowing a comfortable wearing of the processor.

Clinical results were evaluated 1 day, 2 weeks, and 1 month later from surgery. The postoperative follow-up clinically did not show any adverse events. Some cases showed pressure spots because of the head fixation with pressured air pumps. In the first case, the EAR2OS case, this even led to local alopecia that recovered spontaneously after a few months. Case ARCI25_1 suffered from lower back pain at the recovery ward. Although this was a known condition, the length and positioning of RACIS are likely to have provoked this. Again, the lower back pain recovered, but the patient was given additional pain therapy. Case ARCI25_17 suffered from pain on the resolved where the occlusion cuff was placed to register the blood pressure. Again, with an additional painkiller, the patient recovered without permanent injury.

## Discussion

Robotically-assisted cochlear implantation surgery described here enables a surgeon to gain inner ear access in the most standardized fashion. This addresses the main objectives of this first-in-man clinical trial using the HEARO were:

- This system delivers the required intraoperative accuracy of robotic cochlear implantation.
- This system facilitates full manual insertion of the electrode array through the drilled tunnel.

This assistive tool is not designed or it can ever replace a surgeon. It is currently, however, the most autonomous otological tool for CI that meets the true definition of a surgical robot in contrast to many telemanipulators in otology. Parameterization of the anatomy and a radiologically predefined trajectory describing keyhole access with RACIS for CI placement proved safe and efficient in this study. However, this robotic procedure is still highly experimental surgery. Although the device has CE marking, many aspects are susceptible to improvement. The HEARO procedures cover more aspects than can be seen or expected at a first glance. Therefore, it is called a procedure and not a technique. Every aspect in the procedure is an accumulation of knowledge gained by engineered design tested against clinical application and to close the circular learning, it is again evaluated for improvement.

The most essential aspect is safety and pioneering studies have focused on safety and more specifically on not damaging the FN. In 2017, Weber at al. reported the safety of an instrument flight to the “inner ear,” whereas this study actually concentrated on accessing the middle ear starting a voyage toward the inner ear (42). In this series of 6 patients, Caversaccio et al. reported safe access to the middle ear (31) without harm to the FN. From these 6 cases, at least three had an incomplete insertion of the CI in the inner ear. This study never specifically aimed at complete insertion, but rather safe passage through the facial recess. Once the hurdle of facial recess was passed, the surgeon took over manually for the inner ear access and insertion. The surgeon had to create access toward the inner ear through the ear canal with little space. In cases with favorable anatomy, this inner ear access (presumably cochleostomy) could be aligned with the drilled middle ear trajectory. Alignment here refers literally to the inner ear access being on the same line as the middle access, which leads to a straight track. However, it is likely that the manual inner ear access is not aligned and poses major challenges to the surgeons to manipulate the electrode from one track to the other track. The CI array now needs to be manipulated in an S-shaped curve changing from the middle ear trajectory to the manually created inner ear track. There is little room and poor exposure for surgical manipulation, but, moreover, it is highly undesirable because electrodes can easily be damaged due to excessive manipulation. In this study, we did not encounter this problem because the aim of this study was also to drill the inner ear access through the keyhole middle ear access. Consequently, by definition, middle ear and inner ear access tracks were automatically in the same line in continuation of each other. Of course, this required even higher accuracy of the system. Aligning inner ear access with a 1.0-mm diamond burr to enter a 0.8-mm array parallel with the basal turn is less forgiving for inaccuracy as for middle ear access where a 1.8-mm drill diameter needs to pass through a facial recess that is 2.5 mm on average. With only three procedures converted to conventional surgery because of intraoperative inaccuracy, the HEARO system makes a strong claim to provide the required accuracy. It is actually accurate enough to safely warrant middle ear access in 22 from 25 screened cases. Noteworthy, in all these 25 cases, the electrophysiological measurements and estimations have met the safety measurements. It needs further study to reveal, if the radiological safety measures with a required distance of 0.4 mm to the FN are too strict or the electrophysiological measurements need to be stricter.

The inner ear access strategy is an important parameter in personalized inner ear access. Soft surgery principles, one of these strategies, have been discussed and popularized, since 1993 for CI (43). Surgeons are able to perform tissue-preserving approaches, thanks to the widespread hybrid electroacoustic stimulation, thus aiming to preserve hearing. In relation to that, the strategy of access the inner ear affects the angles of cochlear approach (ACA). The ACA affects contact or crash factors regardless of stiffness or stiffness in the sidewall of the basal turn (35). Torres et al. report the “presence of optimal scale axis,” indicating that the semiautomated robot-based system reduces the margin of error in the placement axis (44). In this study, in which Topsakal et al. compared different posterior tympanotomy modalities, they reported that mathematically calculated approaches for RACIS provide the most optimal ACA in an array and a non-crash trajectory that provides easy access to the surgeon (35). The mean in-plane angle in this study is 6.5° and the out-plane angle in this study is 19.0°. Even though angles close to zero might be in favor for electrode placement, the proximity to the FN prevents this ideal trajectory. In other words, 0° angles would pass through the FN or stapes (34). These predetermined “ideal” trajectories cannot be applied clinically because they would pass through the FN (35). In this study, the ideal trajectory to the basal turn is defined as a straight line. This line should be coaxial with the current central axis of the scala tympani to minimize intracochlear damage.

Nevertheless, using the same drilled middle ear access keyhole for inner ear access, it probably explains why all the patients in this study benefited from full insertions of their CI arrays, except for one. Actually, it would be fair to evaluate accuracy not in millimetric distances that deviate from the set target, but in terms of efficiency. With direct linear inner ear access on the same line as middle ear access, the challenge lies more in overcoming the deviations from target perhaps better stated with inaccuracy rather than accuracy. The more we deviate from target, assuming we placed the target in the most ideal spot, the more difficult it will become to insert either with or without manipulations.

In 20 of the 22 cases, RACIS provided efficient inner ear access allowing swift insertions that did not require surgical manipulations. A device that just pushes with a preset force and speed actually could have performed this procedure. In fact, that device will become yet another development in RACIS. In two cases of the performed 22 cases, the provided inner ear access did not allow a swift, correct array placement. Surgical manipulations could not recover the submillimetric inaccuracy in alignment and a manual enlarged RW approach was required for satisfactory insertion.

The reason for not being able to insert could also have been that the target was set wrong. In another case, the diameter of the round window itself was just too small.

Nevertheless, the inability to insert swiftly is inevitably related to the planned trajectory and, therefore, within the responsibilities of the surgeon. Although a surgeon may have been trained in RACIS as a technique, he or she will lack the clinical experience to oversee such problems already in the planning phase. Therefore, it is only fair to consider RACIS an experimental surgery until this knowledge is available and can be shared with trained CI surgeons.

An important aspect of the HEARO procedure is that it facilitates individualized surgery. The dedicated software can screen preoperative scans for eligibility for RACIS. Furthermore, based on patients' individual cochlear parameters, it can estimate the frequency and angle allocation for an individualized selection of the electrode array (36). Individualized surgery proved to be important, especially in anatomically challenging cases. Optimizing such millimetric adjustments requires extensive surgical skill and experience in traditional CI surgery (45). In addition, angle and direction estimation may not be accurate, even by experienced surgeons (46). In a cochlea with an IP-III anomaly, especially the standardized angles of insertion proved important for correct placement. Challenges in this type of case, such as liquor cerebrospinal gusher and electrode misplacement in the internal auditory canal, are well-described in the literature (47, 48). We chose to opt for a cochleostomy rather than a RW approach and even for a shorter array (20 mm) for this patient (case ARCI25_2) because of these shared surgical experiences in literature. In addition, ossifications (postmeningitis case ARCI25_3) and bony alterations around RW because of far advanced otosclerosis (ARCI25_12) are always challenging for conventional surgery because the surgeon needs to rely on experience to find the right angle and to access to the inner ear. Actually, the features to customize insertion angles and target depth encourage us to utilize these aspects of RACIS for the benefit of the patient and surgeon. As a result of measuring the cochlea and the CDL in these patients with RACIS, intervening after determining the anatomical variant of the patient and selecting an appropriate short electrode array reduce the possibility of an incorrect electrode placement. In addition, the insertion of ACA is provided accurately and securely with RACIS. RACIS provides us with the optimum insertion angle by using preoperative planning, intraoperative imaging, anatomical landmarks in malformed anatomical structures, and sometimes using data beyond human perception to guarantee safety and accuracy. In this study, RACIS helped for the planning of the most appropriate cochlear access according to the optimum insertion angles in the patient with IP-III anomaly. Successful application of RACIS to a patient with a cochlear anatomical anomaly for the first time in the literature paved the way for the application of RACIS to patients with different anatomical variations in the future.

The HEARO procedure, which was successfully completed in 22 patients in this study, applies important steps in minimally invasive RACIS. This includes possibilities such as planning the trajectory on the cochlea and personalized inner ear access by configuring the relevant anatomical structures three-dimensionally before surgery. Establishing an autonomous system in the surgical field requires different technologies for the same purpose. But yet, robots can never replace surgeons and there is no such application purpose. Robots are auxiliary surgical instruments that increase the quality and reliability of the surgery. The more complex and diverse tasks a robot has to perform, the more difficult it is to optimize it. Besides that, the more autonomous steps a robot can take in a surgical procedure, the more standard the surgical result can become and the margin of error will be minimized. For this reason, robotic developments in the field of otology have always been followed with amazement and interest by otologists, as it is of great importance to establish submillimetric calculated accuracy and precision. As a result, the robot is superior to a surgeon's dexterity, consistency ability, and surgical acuity. Besides that, anatomical differences and anomalies are difficult areas of robotic surgery for programmers and designers. In addition, intraoperative adverse events, such as unexpected bleeding or unwanted patient movement during anesthesia, cannot be handled by robotic surgery alone nor can the full medicolegal responsibility that comes with surgery be attributed to a robotic device.

We argue that with a newly developed system of robotically-assisted and image-guided approach and FN monitoring, this idea of robotic surgery pushed everyone to develop a complete set of new technologies. This is a turning point because now we are able to do this without any complications in inner ear anomaly patients to get to the precision. Besides, it is not a threat to the surgeon at all. It is like an electric bike or an electric boot: if you do not pedal as a cyclist, it is not going to move forward. Surgeons should reach out to this technology to standardize surgical outcomes in anatomical anomaly and potentially difficult cases and to serve their patients.

## Limitations

There is an obvious learning curve for surgeons, engineers, and nurses performing these surgeries. Therefore, the duration of surgeries was not considered relevant. Time loss occurred due to problems with sterile draping, software hick-ups, poor image resolutions, and absence of routine.

## Conclusion

This study demonstrated the feasibility of RACIS by the HEARO procedure in a clinical study. The accuracy of performing a robotic workflow, more specifically the robotic inner ear access, is reported to meet the current criteria for insertion. In 22 out of 25 patients, a surgeon could complete the HEARO procedure with a full insertion in all the cases, except one where the last electrode was deactivated because it was positioned at the spot of the round window. Future generations of RACIS may focus on improving intraoperative imaging, automated segmentation and trajectory, robotic insertion with controlled speed, and haptic feedback. In addition, smaller tunnels can be developed for smaller electrodes consequently leading to less invasive surgery and more likelihood of preservation of residual hearing levels.

## Data Availability Statement

The raw data supporting the conclusions of this article will be made available by the authors, without undue reservation.

## Ethics Statement

The studies involving human participants were reviewed and approved by Antwerp University Hospital Ethics Committee (EAR2OS: B300201837507 and ARCI25: B300201941457). The patients/participants provided their written informed consent to participate in this study.

## Author Contributions

VT and PH contributed to the conception and design of the study. VT, EH, and AT wrote the first draft of the manuscript. MM and MZ-A codesigned concept and helped with data collection. PG contributed to the data analysis. VV and GM were involved in inclusion and postoperative evaluation of participants. All the authors contributed to manuscript revision and read and approved the submitted version of the manuscript.

## Funding

This study was funded by Department of Otorhinolaryngology Head and Neck Surgery, Antwerp University Hospital receives an unrestrictive grant from MED-EL. VT holds a national FWO FKM senior researcher grant number 18B2222N.

## Conflict of Interest

MM was an employee and shareholder of CASCINATION AG. MZ-A and PG were employees of MED-EL Medical Electronics. The remaining authors declare that the research was conducted in the absence of any commercial or financial relationships that could be construed as a potential conflict of interest.

## Publisher's Note

All claims expressed in this article are solely those of the authors and do not necessarily represent those of their affiliated organizations, or those of the publisher, the editors and the reviewers. Any product that may be evaluated in this article, or claim that may be made by its manufacturer, is not guaranteed or endorsed by the publisher.

## Footnotes

^1^ http://www.earfoundation.org.uk/files/download/1221

## References

[1] Djourno A Eyries C. Auditory prosthesis by means of a distant electrical stimulation of the sensory nerve with the use of an indwelt coiling. Presse Med. (1957) 65:1417. 13484817

[2] Ear Foundation. Cochlear Implants Information Sheet. (2016). Available online at: http://www.earfoundation.org.uk/files/download/1221 (accessed October 29, 2021).

[3] Livingston G Huntley J Sommerlad A Ames D Ballard C Banerjee S. Dementia prevention, intervention, and care: 2020 report of the lancet commission. Lancet. (2020) 396:413–46. 10.1016/S0140-6736(20)30367-6 32738937 PMC7392084

[4] Mertens G Andries E Claes AJ Topsakal V Van de Heyning P Van Rompaey V. Cognitive improvement after cochlear implantation in older adults with severe or profound hearing impairment: a prospective, longitudinal, controlled, multicenter study. Ear Hear. (2020) 42:606–14. 10.1097/AUD.0000000000000962 33055579 PMC8088820

[5] Claes AJ Mertens G Gilles A Hofkens-Van den Brandt A Fransen E Van Rompaey V. The repeatable battery for the assessment of neuropsychological status for hearing impaired individuals (RBANS-H) before and after cochlear implantation: a protocol for a prospective, longitudinal cohort study. Front Neurosci. (2016) 10:512. 10.3389/fnins.2016.00512 27895549 PMC5108794

[6] Van de Heyning P Atlas M Baumgartner W-D Caversaccio M Gavilan J Godey B.. The reliability of hearing implants: report on the type and incidence of cochlear implant failures. Cochlear Implants Int. (2020) 21:228–37. 10.1080/14670100.2020.1735678 32156201

[7] Dhanasingh A. Jolly C. An overview of cochlear implant electrode array designs. Hear Res. (2017) 356:93–103. 10.1016/j.heares.2017.10.005 29102129

[8] Wouters J McDermott HJ Francart T. Sound coding in cochlear implants: from electric pulses to hearing. IEEE Signal Process Mag. (2015) 32:67–80. 10.1109/MSP.2014.2371671

[9] House WF. Cochlear implants. Ann Otol Rhinol Laryngol. (1976) 85:1–93. 10.1177/00034894760850S301 779582

[10] Kiratzidis T Arnold W Iliades T. Veria operation updated. I The trans-canal wall cochlear implantation. ORL J Otorhinolaryngol Relat Spec. (2002) 64:406–12. 10.1159/000067578 12499764

[11] Kronenberg J Migirov L. Dagan T. Suprameatal approach: new surgical approach for cochlear implantation. J Laryngol Otol. (2001) 115:283–5. 10.1258/0022215011907451 11276329

[12] Häusler R. Cochlear implantation without mastoidectomy: the pericanal electrode insertion technique. Acta Otolaryngol. (2002) 122:715–9. 10.1080/00016480260349773 12484647

[13] Bruijnzeel H Ziylan F Cattani G Grolman W. Topsakal V. Retrospective complication rate comparison between surgical techniques in paediatric cochlear implantation. Clin Otolaryngol. (2016) 41:666–72. 10.1111/coa.12582 26541783

[14] Havenith S Lammers MJW Tange RA Trabalzini F della Volpe A van der Heijden GJMG. Hearing preservation surgery: cochleostomy or round window approach? a systematic review. Otol Neurotol. (2013) 34:667–74. 10.1097/MAO.0b013e318288643e 23640087

[15] Vashishth A Fulcheri A Prasad SC Bassi M Rossi G Caruso A.. Cochlear implantation in cochlear ossification: retrospective review of etiologies, surgical considerations, and auditory outcomes. Otol Neurotol. (2018) 39:17–28. 10.1097/MAO.0000000000001613 29065093

[16] Sennaroglu L. Cochlear implantation in inner ear malformations-a review article. Cochlear Implants Int. (2010) 11:4–41. 10.1002/cii.416 19358145

[17] Miranda PC Sampaio AL Lopes RA Ramos Venosa A de Oliveira CA. Hearing preservation in cochlear implant surgery. Int J Otolaryngol. (2014) 2014:468515. 10.1155/2014/468515 25276136 PMC4167950

[18] Dalbert A Pfiffner F Hoesli M Koka K Veraguth D Roosli C. Assessment of cochlear function during cochlear implantation by extra- and intracochlear electrocochleography. Front Neurosci. (2018) 12:18. 10.3389/fnins.2018.00018 29434534 PMC5790789

[19] Paasche G Bockel F Tasche C Lesinski-Schiedat A Lenarz T. Changes of postoperative impedances in cochlear implant patients: the short-term effects of modified electrode surfaces and intracochlear corticosteroids. Otol Neurotol. (2006) 27:639–47. 10.1097/01.mao.0000227662.88840.61 16868511

[20] Majdani O Rau TS Baron S Eilers H Baier C Heimann B. A robot-guided minimally invasive approach for cochlear implant surgery: preliminary results of a temporal bone study. Int J Comput Assist Radiol Surg. (2009) 4:475–86. 10.1007/s11548-009-0360-8 20033531

[21] Müller S Kahrs LA Gaa J Tauscher S Kluge M John S.. Workflow assessment as a preclinical development tool: Surgical process models of three techniques for minimally invasive cochlear implantation. Int J Comput Assist Radiol Surg. (2019) 14:1389–401. 10.1007/s11548-019-02002-3 31168671

[22] Labadie RF Balachandran R Noble JH Blachon GS Mitchell JE Reda FA. Minimally invasive image-guided cochlear implantation surgery: first report of clinical implementation. Laryngoscope. (2014) 124:1915–22. 10.1002/lary.24520 24272427 PMC4453761

[23] Caversaccio M Gavaghan K Wimmer W Williamson T Ansò J Mantokoudis G. Robotic cochlear implantation: surgical procedure and first clinical experience. Acta Otolaryngol. (2017) 137:447–54. 10.1080/00016489.2017.1278573 28145157

[24] Labadie RF Chodhury P Cetinkaya E Balachandran R Haynes DS Fenlon MR. Minimally invasive, image-guided, facial-recess approach to the middle ear: demonstration of the concept of percutaneous cochlear access *in vitro*. Otol Neurotol. (2005) 26:557–62. 10.1097/01.mao.0000178117.61537.5b 16015146

[25] Schipper J Klenzner T Aschendorff A Arapakis I Ridder GJ Laszig R. Navigiert-kontrollierte Kochleostomie. Ist eine Verbesserung der Ergebnisqualität in der Kochleaimplantatchirurgie möglich? [Navigation-controlled cochleostomy Is an improvement in the quality of results for cochlear implant surgery possible?]. HNO. (2004) 52:329–35. 10.1007/s00106-004-1057-5 15014891

[26] Ansó J Dür C Apelt M Venail F Scheidegger O Seidel K. Prospective validation of facial nerve monitoring to prevent nerve damage during robotic drilling. Front Surg. (2019) 6:58. 10.3389/fsurg.2019.00058 31632981 PMC6781655

[27] Labadie RF Balachandran R Mitchell JE Noble JH Majdani O Haynes DS. Clinical validation study of percutaneous cochlear access using patient-customized microstereotactic frames. Otol Neurotol. (2010) 31:94–9. 10.1097/MAO.0b013e3181c2f81a 20019561 PMC2845321

[28] Warren FM Balachandran R Fitzpatrick JM Labadie RF. Percutaneous cochlear access using bone-mounted, customized drill guides: demonstration of concept *in vitro*. Otol Neurotol. (2007) 28:325–9. 10.1097/01.mao.0000253287.86737.2e 17414037

[29] Baron S Eilers H Munske B Toennies JL Balachandran R Labadie RF. Percutaneous inner-ear access *via* an image-guided industrial robot system. Proc Inst Mech Eng H. (2010) 224:633–49. 10.1243/09544119JEIM781 20718268 PMC4107213

[30] Klenzner T Ngan CC Knapp FB Knoop H Kromeier J Aschendorff A. New strategies for high precision surgery of the temporal bone using a robotic approach for cochlear implantation. Eur Arch Otorhinolaryngol. (2009) 266:955–60. 10.1007/s00405-008-0825-3 19015866

[31] Caversaccio M Wimmer W Anso J Mantokoudis G Gerber N Rathgeb C. Robotic middle ear access for cochlear implantation: First in man. PLoS ONE. (2019) 14:e0220543. 10.1371/journal.pone.0220543 31374092 PMC6677292

[32] Coulson CJ Assadi MZ Taylor RP Du X Brett PN Reid AP. A smart micro-drill for cochleostomy formation: a comparison of cochlear disturbances with manual drilling and a human trial. Cochlear Implants Int. (2013) 14:98–106. 10.1179/1754762811Y.0000000018 22333534

[33] Atturo F Barbara M Rask-Andersen H. Is the human round window really round? an anatomic study with surgical implications. Otol Neurotol. (2014) 35:1354–60. 10.1097/MAO.0000000000000332 24608377

[34] Wimmer W Venail F Williamson T Akkari M Gerber N Weber S. Semiautomatic cochleostomy target and insertion trajectory planning for minimally invasive cochlear implantation. Biomed Res Int. (2014) 2014:596498. 10.1155/2014/596498 25101289 PMC4101975

[35] Topsakal V Matulic M Assadi MZ Mertens G Rompaey VV Van de Heyning P. Comparison of the surgical techniques and robotic techniques for cochlear implantation in terms of the trajectories toward the inner ear. J Int Adv Otol. (2020) 16:3–7. 10.5152/iao.2020.8113 32209514 PMC7224420

[36] Mertens G Van Rompaey V Van de Heyning P Gorris E Topsakal V. Prediction of the cochlear implant electrode insertion depth: clinical applicability of two analytical cochlear models. Sci Rep. (2020) 10:3340. 10.1038/s41598-020-58648-6 32094372 PMC7039896

[37] Donkelaar HJT Elliott KL Fritzsch B Kachlik D Carlson M Isaacson B. An updated terminology for the internal ear with combined anatomical and clinical terms. J Phonet Audiol. (2020) 6:147. 10.35248/2471-9455.20.6.147

[38] Ten Donkelaar HJ Kachlík D Tubbs RS. An Illustrated Terminologia Neuroanatomica. Cham: Springer International Publishing (2018).

[39] Topsakal V Kachlik D Bahşi I Carlson M Isaacson B Broman J. Relevant temporal bone anatomy for robotic cochlear implantation: an updated terminology combined with anatomical and clinical terms. Transl Res Anat. (2021) 25:100138. 10.1016/j.tria.2021.100138

[40] Tekin AM Matulic M Wuyts W Assadi MZ Mertens G Rompaey VV. A new pathogenic variant in POU3F4 causing deafness due to an incomplete partition of the cochlea paved the way for innovative surgery. Genes. (2021) 12:613. 10.3390/genes12050613 33919129 PMC8143104

[41] Meyerhoff WL Stringer SP Roland PS. Rambo procedure: modification and application. Laryngoscope. (1988) 98:795–6. 10.1288/00005537-198807000-00025 3386390

[42] Weber S Gavaghan K Wimmer W Williamson T Gerber N Anso J. Instrument flight to the inner ear. Sci Robot. (2017) 2:eaal4916. 10.1126/scirobotics.aal4916 30246168 PMC6150423

[43] Lehnhardt E. Intrakochleäre Plazierung der Cochlear-Implant-Elektroden in soft surgery technique [Intracochlear placement of cochlear implant electrodes in soft surgery technique]. HNO. (1993) 41:356–9. 8376183

[44] Torres R Kazmitcheff G De Seta D Ferrary E Sterkers O Nguyen Y. Improvement of the insertion axis for cochlear implantation with a robot-based system. Eur Arch Otorhinolaryngol. (2017) 274:715–21. 10.1007/s00405-016-4329-2 27704279

[45] Incesulu A Adapinar B Kecik C. Cochlear implantation in cases with incomplete partition type III (X-linked anomaly). Eur Arch Otorhinolaryngol. (2008) 265:1425–30. 10.1007/s00405-008-0614-z 18305951

[46] Torres R Kazmitcheff G Bernardeschi D De Seta D Bensimon JL Ferrary E. Variability of the mental representation of the cochlear anatomy during cochlear implantation. Eur Arch Otorhinolaryngol. (2016) 273:2009–18. 10.1007/s00405-015-3763-x 26324880

[47] Sennaroglu L Bajin MD. Incomplete partition type III: a rare and difficult cochlear implant surgical indication. Auris Nasus Larynx. (2018) 45:26–32. 10.1016/j.anl.2017.02.006 28318810

[48] Saeed H Powell HRF Saeed SR. Cochlear implantation in X-linked deafness - how to manage the surgical challenges. Cochlear Implants Int. (2016) 17:178–83. 10.1080/14670100.2016.1180018 27142359

